# Constructing a meaningful evolutionary average at the phylogenetic center of mass

**DOI:** 10.1186/1471-2105-8-222

**Published:** 2007-06-26

**Authors:** Eric A Stone, Arend Sidow

**Affiliations:** 1 Bioinformatics Research Center, North Carolina State University, Raleigh, NC 27695-7566, USA; 2 Department of Statistics, North Carolina State University, Raleigh, NC 27695-8203, USA; 3 Department of Pathology, Stanford University, Stanford, CA 94305-5324, USA; 4 Department of Genetics, Stanford University, Stanford, CA 94305-5120, USA

## Abstract

**Background:**

As a consequence of the evolutionary process, data collected from related species tend to be similar. This similarity by descent can obscure subtler signals in the data such as the evidence of constraint on variation due to shared selective pressures. In comparative sequence analysis, for example, sequence similarity is often used to illuminate important regions of the genome, but if the comparison is between closely related species, then similarity is the rule rather than the interesting exception. Furthermore, and perhaps worse yet, the contribution of a divergent third species may be masked by the strong similarity between the other two. Here we propose a remedy that weighs the contribution of each species according to its phylogenetic placement.

**Results:**

We first solve the problem of summarizing data related by phylogeny, and we explain why an average should operate on the entire evolutionary trajectory that relates the data. This perspective leads to a new approach in which we define the average in terms of the phylogeny, using the data and a stochastic model to obtain a probability on evolutionary trajectories. With the assumption that the data evolve according to a Brownian motion process on the tree, we show that our evolutionary average can be computed as convex combination of the species data. Thus, our approach, called the BranchManager, defines both an average and a novel taxon weighting scheme. We compare the BranchManager to two other methods, demonstrating why it exhibits desirable properties. In doing so, we devise a framework for comparison and introduce the concept of a representative point at which the average is situated.

**Conclusion:**

The BranchManager uses as its representative point the phylogenetic center of mass, a choice which has both intuitive and practical appeal. Because our average is intrinsic to both the dataset and to the phylogeny, we expect it and its corresponding weighting scheme to be useful in all sorts of studies where interspecies data need to be combined. Obvious applications include evolutionary studies of morphology, physiology or behaviour, but quantitative measures such as sequence hydrophobicity and gene expression level are amenable to our approach as well. Other areas of potential impact include motif discovery and vaccine design. A Java implementation of the BranchManager is available for download, as is a script written in the statistical language R.

## Background

Over the past few decades, there has been a groundswell of support for phylogenetic methods to account for the ancestral relationships between interspecies data [1]. It is apparent that the signal of similarity by descent is sufficiently strong to mask other potentially interesting patterns in evolutionary samples [2]; as a result, researchers have developed a suite of approaches for mitigating the influence of relatedness on inference from species or sequence data [3-10]. The most sophisticated approaches directly use phylogeny in an inferential procedure, and when this is possible, the explicit incorporation of an evolutionary model seems the best and most logical choice [11,12]. Nevertheless, in many applications, no model readily presents itself, and the task instead reduces to modifying the impact of each species or sequence datum to reflect the unique contribution of that datum to the breadth included in the study. Disagreement on the quantitative interpretation of this qualitative endpoint has led to a number of competing methods [3-10], and as the goals of these methods differ, there is no direct means of comparison [13].

How to average data collected from related taxa may be the most basic problem in which the vagaries of phylogenetic relationships come into play. Because of the dependence structure imposed by the phylogenetic tree, it is inappropriate to treat sequences or other data collected from extant species as independent [14]. When the tree is known or can be confidently inferred, it provides the raw material to tease apart the relative contributions of each species to an average. Still, even after agreeing on an appropriate dependence structure, it remains to define what qualities of the data the average should reflect. Put another way, what defines the "average" parameter to be estimated from the species data?

Typically, the process of averaging sample data creates a statistic that is used to estimate a population parameter. But, when the process is averaging data related by phylogeny, neither the population nor the parameter of interest is clear. One exception to this uncertainty is the ancestral reconstruction of a trait (e.g. phenotype, genotype etc.), which can be viewed as a type of averaging in which the parameter of interest is the unknown value of the most recent common ancestor of the set of species in the study. In that case, the population can be taken as the hypothetical collection of values of all species descended from the common ancestor of the species in the sample. This approach was taken in [3], and, in concert with a evolutionary model, yields a formal estimation procedure. Specifically, by assuming that evolution of a trait from the common ancestral value proceeds forward in time according to a Brownian motion on the phylogeny [15,16], the probability of observing the sample species data *X*_1 _.... *X*_*n *_can be expressed in terms of the unknown ancestral value *μ *as

Pr⁡(X1,…,Xn|μ)=1(2π)n|Σ|exp⁡(−12(X−μ1)TΣ−1(X−μ1))
 MathType@MTEF@5@5@+=feaafiart1ev1aaatCvAUfKttLearuWrP9MDH5MBPbIqV92AaeXatLxBI9gBaebbnrfifHhDYfgasaacH8akY=wiFfYdH8Gipec8Eeeu0xXdbba9frFj0=OqFfea0dXdd9vqai=hGuQ8kuc9pgc9s8qqaq=dirpe0xb9q8qiLsFr0=vr0=vr0dc8meaabaqaciaacaGaaeqabaqabeGadaaakeaacyGGqbaucqGGYbGCdaqadaqaaiabdIfaynaaBaaaleaacqaIXaqmaeqaaOGaeiilaWIaeSOjGSKaeiilaWIaemiwaG1aaSbaaSqaaiabd6gaUbqabaGccqGG8baFiiGacqWF8oqBaiaawIcacaGLPaaacqGH9aqpdaWcaaqaaiabigdaXaqaamaakaaabaWaaeWaaeaacqaIYaGmcqWFapaCaiaawIcacaGLPaaadaahaaWcbeqaaiabd6gaUbaakmaaemaabaGaeu4OdmfacaGLhWUaayjcSdaaleqaaaaakiGbcwgaLjabcIha4jabcchaWnaabmaabaGaeyOeI0YaaSaaaeaacqaIXaqmaeaacqaIYaGmaaWaaeWaaeaacqWHybawcqGHsislcqWF8oqBcqWHXaqmaiaawIcacaGLPaaadaahaaWcbeqaaiabdsfaubaakiabfo6atnaaCaaaleqabaGaeyOeI0IaeGymaedaaOWaaeWaaeaacqWHybawcqGHsislcqWF8oqBcqWHXaqmaiaawIcacaGLPaaaaiaawIcacaGLPaaaaaa@62FF@

where **X **is the length-*n *column vector of species data, **1 **is a length-*n *column vector of ones, and Σ is the covariance matrix specified by the phylogeny under the model (see Methods and Additional file 1). The parameter of interest *μ *can then be estimated by maximum likelihood as μ^=wTX=1TΣ−1X1TΣ−11
 MathType@MTEF@5@5@+=feaafiart1ev1aaatCvAUfKttLearuWrP9MDH5MBPbIqV92AaeXatLxBI9gBaebbnrfifHhDYfgasaacH8akY=wiFfYdH8Gipec8Eeeu0xXdbba9frFj0=OqFfea0dXdd9vqai=hGuQ8kuc9pgc9s8qqaq=dirpe0xb9q8qiLsFr0=vr0=vr0dc8meaabaqaciaacaGaaeqabaqabeGadaaakeaaiiGacuWF8oqBgaqcaiabg2da9iabhEha3naaCaaaleqabaGaemivaqfaaOGaeCiwaGLaeyypa0ZaaSaaaeaacqWHXaqmdaahaaWcbeqaaiabdsfaubaakiabfo6atnaaCaaaleqabaGaeyOeI0IaeGymaedaaOGaeCiwaGfabaGaeCymaeZaaWbaaSqabeaacqWGubavaaGccqqHJoWudaahaaWcbeqaaiabgkHiTiabigdaXaaakiabhgdaXaaaaaa@42BF@, which doubles as a recipe for constructing a weighted average of the data **X **collected from related species. Importantly, however, this average is designed to faithfully estimate the *ancestral *data, and depending on the shape of the phylogeny, the most recent common ancestor may be far from representative of taxonomic distribution of the species in the study. A simple alternative might be to find a point on the phylogeny that does appropriately represent the taxonomic distribution; for example, this rationale has motivated the center-of-tree approach as a remedy for phylogenetic deficiencies in consensus and ancestral-state HIV vaccine design [17,18]. Unfortunately, this exchanges one problem for another, as in general no clear concept of a representative point currently exists.

Toward the same goal of representing the whole of the phylogeny, we turn to a different interpretation of the estimation problem in [3] and develop a useful alternative in similar spirit. In what follows, we redefine the concept of a phylogenetic average, comparing this in philosophy to existing methods. In doing so, we show that our notion of an average is inherently robust against the vagaries of ancestral relationships, and we discuss how this directly relates to the use of species weights. Additionally, we show that our approach maps the average value to a representative point in species space that lies off the phylogeny. This last result provides an alternative to ancestral reconstruction that may in some cases be preferable, including when the goal of reconstruction is vaccine design.

## Results

### Defining an average

The Brownian motion model has traditionally been used to describe the evolution of quantitative traits undergoing genetic drift [15,16]. As in the averaging context of [3], we interpret the Brownian motion as random flux about an optimum, and we consider the effects of variation about that optimum to be negligible. Furthermore, there exists a prototypical value *μ*, which in [3] was attained by the ancestral species at the phylogenetic root. In our approach, we also seek a prototypical *μ*, but instead make no a priori assumption that it is the ancestral species which attains the value of interest. Unfortunately, by no longer treating the root as a representative point, both the biological and statistical interpretations of *μ *in [3] are lost. To construct a meaningful parameter in this setting, our approach is to consider the sample paths of the stochastic process from which the data are presumed to arise.

When data are collected from related taxa, only the endpoints of the evolutionary process are observed. But suppose we adopt a "missing data" perspective and consider the case when the entire evolutionary trajectory is known. Under this scenario, there is no "averaging problem", because we can directly compute the average of the values observed over the course of the evolutionary trajectory. The question then is how to estimate this same average in the absence of the missing data. We propose an average of averages, namely the expectation of the average trajectory value with respect to a probability measure on all possible trajectories. It turns out that this can be easily accomplished within the existing Brownian motion framework.

### Computing an average

Recall that the model of [3] views the species data *X*_1 _..., *X*_*n *_as the multivariate endpoint of a Brownian motion on the phylogeny. The initial value of the process is the unknown parameter *μ *at the root, which we will henceforth call *X*_*A *_to reflect its meaning as the unobserved ancestral datum. We begin this section by exploiting the reversibility of the model to remove the unnatural emphasis on the root and its value *X*_*A*_.

**Figure 1:**
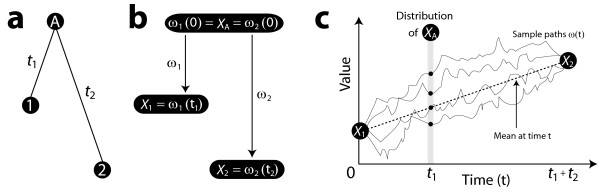
Reparameterizing Brownian motion on a two-taxon phylogeny. (a) Species A is the most recent common ancestor of species 1 and 2. Their respective divergence times are t_1 _and t_2_. (b) The observed data *X*_1 _and *X*_2 _are assumed to have evolved independently from the ancestral value *X*_*A *_according to lineage-specific Brownian motion processes *ω*_1 _and *ω*_2_. (c) Considering the species data to be fixed and the ancestral datum to be random, the lineage-specific Brownian motions can be combined into one process *ω*, essentially running up one branch and then down the other. The *x-*axis is a reparameterization of time in (b) such that *X*_1 _is the value of the process at time 0 while *X*_2 _is the value of the process at time t_1 _+ t_2_. The value of the process at time t_1 _is the unknown quantity *X*_*A*_, whose distribution is determined by the behaviour of the Brownian bridge obtained by conditioning a Brownian motion on its endpoints. Four representative sample paths of the Brownian bridge *ω*(t) are shown; for each, the value of *X*_*A *_is *ω*(t_1_) as indicated by a solid black dot. The mean of the process at any fixed time t is given by the dashed line, which in the case of t = t_1 _corresponds to the mean of the distribution of *X*_*A*_.

To develop our ideas, it is easiest to proceed using a two-taxon tree for which the data are simply *X*_1 _and *X*_2 _(Figure 1a). The tree relates two extant species, labelled 1 and 2, whose data are *X*_1 _and *X*_2_, respectively. There are t_1 _units of evolutionary ivergence separating species 1 and the ancestral species A, while species 2 and the ancestral species A are separated by t_2 _units. (To avoid confusion, we will take quantities such as t_1 _and t_2 _to be generic branch lengths whose units can be either times or distances.) The Brownian motion model assumes that *X*_1 _and *X*_2 _have evolved from the ancestral value *X*_*A *_according to lineage-specific Brownian motion processes *ω*_1 _and *ω*_2 _(Figure 1b). The two independent processes begin at time 0 at the ancestral species A, with *ω*_1_(0) = *ω*_2_(0) = *X*_*A*_. Running forward in time, the data are assumed to be the endpoints of these processes: *ω*_1_(t_1_) = *X*_1 _and *ω*_2_(t_2_) = *X*_2_. The approach of [3] views the species data as random and the ancestral value as an estimable unknown: conditional on *X*_*A*_, *X*_1_, and *X*_2 _are independent Normal random variables with common mean *X*_*A *_and variances t_1 _and t_2_, respectively. By contrast, we advocate conditioning on the species data and considering the evolutionary trajectory (including *X*_*A *_and all other ancestral values) as random. Among other benefits, this approach allows us to exploit the reversibility of Brownian motion, combining *ω*_1 _and *ω*_2 _into one process *ω *(Figure 1c). According to the model, the combined process *ω *is a Brownian bridge, or in other words, a Brownian motion conditioned on both of its endpoints [19]. The distribution of the now random *X*_*A *_is simply the distribution of *ω*(t_1_), which theory gives to be Normal with mean 1t1+t2(t1X1+t2X2)
 MathType@MTEF@5@5@+=feaafiart1ev1aaatCvAUfKttLearuWrP9MDH5MBPbIqV92AaeXatLxBI9gBaebbnrfifHhDYfgasaacH8akY=wiFfYdH8Gipec8Eeeu0xXdbba9frFj0=OqFfea0dXdd9vqai=hGuQ8kuc9pgc9s8qqaq=dirpe0xb9q8qiLsFr0=vr0=vr0dc8meaabaqaciaacaGaaeqabaqabeGadaaakeaadaWcaaqaaiabigdaXaqaaiabdsha0naaBaaaleaacqaIXaqmaeqaaOGaey4kaSIaemiDaq3aaSbaaSqaaiabikdaYaqabaaaaOWaaeWaaeaacqWG0baDdaWgaaWcbaGaeGymaedabeaakiabdIfaynaaBaaaleaacqaIXaqmaeqaaOGaey4kaSIaemiDaq3aaSbaaSqaaiabikdaYaqabaGccqWGybawdaWgaaWcbaGaeGOmaidabeaaaOGaayjkaiaawMcaaaaa@4019@ and variance t1t2t1+t2
 MathType@MTEF@5@5@+=feaafiart1ev1aaatCvAUfKttLearuWrP9MDH5MBPbIqV92AaeXatLxBI9gBaebbnrfifHhDYfgasaacH8akY=wiFfYdH8Gipec8Eeeu0xXdbba9frFj0=OqFfea0dXdd9vqai=hGuQ8kuc9pgc9s8qqaq=dirpe0xb9q8qiLsFr0=vr0=vr0dc8meaabaqaciaacaGaaeqabaqabeGadaaakeaadaWcaaqaaiabdsha0naaBaaaleaacqaIXaqmaeqaaOGaemiDaq3aaSbaaSqaaiabikdaYaqabaaakeaacqWG0baDdaWgaaWcbaGaeGymaedabeaakiabgUcaRiabdsha0naaBaaaleaacqaIYaGmaeqaaaaaaaa@37F4@ (Figure 1c). In particular, the mean of this distribution is exactly the estimated average μ^
 MathType@MTEF@5@5@+=feaafiart1ev1aaatCvAUfKttLearuWrP9MDH5MBPbIqV92AaeXatLxBI9gBaebbnrfifHhDYfgasaacH8akY=wiFfYdH8Gipec8Eeeu0xXdbba9frFj0=OqFfea0dXdd9vqai=hGuQ8kuc9pgc9s8qqaq=dirpe0xb9q8qiLsFr0=vr0=vr0dc8meaabaqaciaacaGaaeqabaqabeGadaaakeaaiiGacuWF8oqBgaqcaaaa@2E79@ from [3].

**Figure 2:**
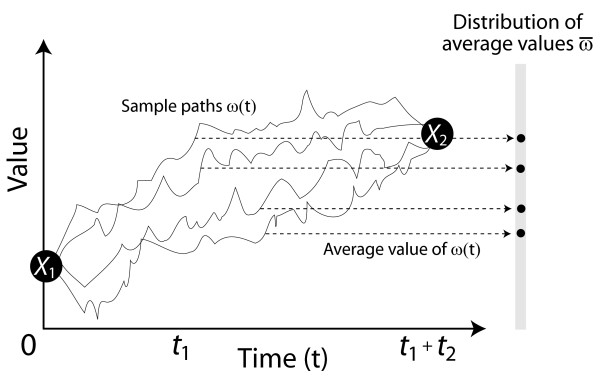
A distribution of averages. In Figure 1c, each sample path *ω*(t) was evaluated at t_1 _to obtain a value of *X*_*A*_. The average proposed in [3] is the mean of the distribution of these *X*_*A *_values. In this figure, the average value ω¯
 MathType@MTEF@5@5@+=feaafiart1ev1aaatCvAUfKttLearuWrP9MDH5MBPbIqV92AaeXatLxBI9gBaebbnrfifHhDYfgasaacH8akY=wiFfYdH8Gipec8Eeeu0xXdbba9frFj0=OqFfea0dXdd9vqai=hGuQ8kuc9pgc9s8qqaq=dirpe0xb9q8qiLsFr0=vr0=vr0dc8meaabaqaciaacaGaaeqabaqabeGadaaakeaaiiGacuWFjpWDgaqeaaaa@2E98@ of each sample path is computed using the formula ω¯=1t1+t2∫0t1+t2ω(t)dt
 MathType@MTEF@5@5@+=feaafiart1ev1aaatCvAUfKttLearuWrP9MDH5MBPbIqV92AaeXatLxBI9gBaebbnrfifHhDYfgasaacH8akY=wiFfYdH8Gipec8Eeeu0xXdbba9frFj0=OqFfea0dXdd9vqai=hGuQ8kuc9pgc9s8qqaq=dirpe0xb9q8qiLsFr0=vr0=vr0dc8meaabaqaciaacaGaaeqabaqabeGadaaakeaaiiGacuWFjpWDgaqeaGGaaiab+1da9maalaaabaGaeGymaedabaGaemiDaq3aaSbaaSqaaiabigdaXaqabaGccqGHRaWkcqWG0baDdaWgaaWcbaGaeGOmaidabeaaaaGcdaWdXaqaaiab=L8a3naabmaabaGaemiDaqhacaGLOaGaayzkaaaaleaacqaIWaamaeaacqWG0baDdaWgaaadbaGaeGymaedabeaaliabgUcaRiabdsha0naaBaaameaacqaIYaGmaeqaaaqdcqGHRiI8aOGaemizaqMaemiDaqhaaa@4762@. The mean *μ *of the distribution of these values is the average that we propose here.

Earlier we proposed to average the values observed over the course of the evolutionary trajectory; now we have a framework under which this can be accomplished. Rather than take *ω*(t_1_) to be the quantity of interest for each sample path, we consider for each sample path its average ω¯=1t1+t2∫0t1+t2ω(t)dt
 MathType@MTEF@5@5@+=feaafiart1ev1aaatCvAUfKttLearuWrP9MDH5MBPbIqV92AaeXatLxBI9gBaebbnrfifHhDYfgasaacH8akY=wiFfYdH8Gipec8Eeeu0xXdbba9frFj0=OqFfea0dXdd9vqai=hGuQ8kuc9pgc9s8qqaq=dirpe0xb9q8qiLsFr0=vr0=vr0dc8meaabaqaciaacaGaaeqabaqabeGadaaakeaaiiGacuWFjpWDgaqeaGGaaiab+1da9maalaaabaGaeGymaedabaGaemiDaq3aaSbaaSqaaiabigdaXaqabaGccqGHRaWkcqWG0baDdaWgaaWcbaGaeGOmaidabeaaaaGcdaWdXaqaaiab=L8a3naabmaabaGaemiDaqhacaGLOaGaayzkaaaaleaacqaIWaamaeaacqWG0baDdaWgaaadbaGaeGymaedabeaaliabgUcaRiabdsha0naaBaaameaacqaIYaGmaeqaaaqdcqGHRiI8aOGaemizaqMaemiDaqhaaa@4762@ (Figure 2).

The Brownian motion model induces a distribution on ω¯
 MathType@MTEF@5@5@+=feaafiart1ev1aaatCvAUfKttLearuWrP9MDH5MBPbIqV92AaeXatLxBI9gBaebbnrfifHhDYfgasaacH8akY=wiFfYdH8Gipec8Eeeu0xXdbba9frFj0=OqFfea0dXdd9vqai=hGuQ8kuc9pgc9s8qqaq=dirpe0xb9q8qiLsFr0=vr0=vr0dc8meaabaqaciaacaGaaeqabaqabeGadaaakeaaiiGacuWFjpWDgaqeaaaa@2E98@, and the mean of this distribution is exactly the average of averages we seek. Using Fubini's Theorem and a generalization of the result for the distribution of *ω*(t_1_), we have

E[ω¯] =1t1+t2∫0t1+t2E[ω(t)]dt=1t1+t2∫0t1+t21t1+t2[tX1+(t1+t2−t)X2]dt=12[X1+X2].
 MathType@MTEF@5@5@+=feaafiart1ev1aaatCvAUfKttLearuWrP9MDH5MBPbIqV92AaeXatLxBI9gBaebbnrfifHhDYfgasaacH8akY=wiFfYdH8Gipec8Eeeu0xXdbba9frFj0=OqFfea0dXdd9vqai=hGuQ8kuc9pgc9s8qqaq=dirpe0xb9q8qiLsFr0=vr0=vr0dc8meaabaqaciaacaGaaeqabaqabeGadaaakeaacqWGfbqrdaWadaqaaGGaciqb=L8a3zaaraaacaGLBbGaayzxaaGaeeiiaaIaeeypa0ZaaSaaaeaacqaIXaqmaeaacqWG0baDdaWgaaWcbaGaeGymaedabeaakiabgUcaRiabdsha0naaBaaaleaacqaIYaGmaeqaaaaakmaapedabaGaemyrau0aamWaaeaacqWFjpWDdaqadaqaaiabdsha0bGaayjkaiaawMcaaaGaay5waiaaw2faaaWcbaGaeGimaadabaGaemiDaq3aaSbaaWqaaiabigdaXaqabaWccqGHRaWkcqWG0baDdaWgaaadbaGaeGOmaidabeaaa0Gaey4kIipakiabdsgaKjabdsha0jabg2da9maalaaabaGaeGymaedabaGaemiDaq3aaSbaaSqaaiabigdaXaqabaGccqGHRaWkcqWG0baDdaWgaaWcbaGaeGOmaidabeaaaaGcdaWdXaqaamaalaaabaGaeGymaedabaGaemiDaq3aaSbaaSqaaiabigdaXaqabaGccqGHRaWkcqWG0baDdaWgaaWcbaGaeGOmaidabeaaaaGcdaWadaqaaiabdsha0jabdIfaynaaBaaaleaacqaIXaqmaeqaaOGaey4kaSYaaeWaaeaacqWG0baDdaWgaaWcbaGaeGymaedabeaakiabgUcaRiabdsha0naaBaaaleaacqaIYaGmaeqaaOGaeyOeI0IaemiDaqhacaGLOaGaayzkaaGaemiwaG1aaSbaaSqaaiabikdaYaqabaaakiaawUfacaGLDbaaaSqaaiabicdaWaqaaiabdsha0naaBaaameaacqaIXaqmaeqaaSGaey4kaSIaemiDaq3aaSbaaWqaaiabikdaYaqabaaaniabgUIiYdGccqWGKbazcqWG0baDcqGH9aqpdaWcaaqaaiabigdaXaqaaiabikdaYaaadaWadaqaaiabdIfaynaaBaaaleaacqaIXaqmaeqaaOGaey4kaSIaemiwaG1aaSbaaSqaaiabikdaYaqabaaakiaawUfacaGLDbaacqGGUaGlaaa@87B2@

In words: when there are only two data points, our method dictates that they have equal weight in an average. The mathematical discourse runs the danger of obscuring what has transpired: in computing the average of averages, Fubini's Theorem gave the choice of which average to take first. Averaging first by time point rather than by sample path, we were able to collapse the distribution of sample paths into the dashed line of means (Figure 1c). Taking the average of that straight line on the *y*-axis was trivial and yielded the halfway point 12[X1+X2].
 MathType@MTEF@5@5@+=feaafiart1ev1aaatCvAUfKttLearuWrP9MDH5MBPbIqV92AaeXatLxBI9gBaebbnrfifHhDYfgasaacH8akY=wiFfYdH8Gipec8Eeeu0xXdbba9frFj0=OqFfea0dXdd9vqai=hGuQ8kuc9pgc9s8qqaq=dirpe0xb9q8qiLsFr0=vr0=vr0dc8meaabaqaciaacaGaaeqabaqabeGadaaakeaadaWcaaqaaiabigdaXaqaaiabikdaYaaadaWadaqaaiabdIfaynaaBaaaleaacqaIXaqmaeqaaOGaey4kaSIaemiwaG1aaSbaaSqaaiabikdaYaqabaaakiaawUfacaGLDbaacqGGUaGlaaa@3716@ This is also the mean of the distribution of average values shown in Figure 2.

While a two-taxon application is trivial, the methods outlined above are completely general and apply to datasets and phylogenies of arbitrary size. Our average of averages is easily computed for any tree topology, binary or multifurcating, without regard to the placement of the phylogenetic root. In the two-taxon case, we saw that our result took the form of a weighted average. This is also general; under the Brownian motion model, the average of averages will always be a convex combination wTX=∑i=1nwiXi
 MathType@MTEF@5@5@+=feaafiart1ev1aaatCvAUfKttLearuWrP9MDH5MBPbIqV92AaeXatLxBI9gBaebbnrfifHhDYfgasaacH8akY=wiFfYdH8Gipec8Eeeu0xXdbba9frFj0=OqFfea0dXdd9vqai=hGuQ8kuc9pgc9s8qqaq=dirpe0xb9q8qiLsFr0=vr0=vr0dc8meaabaqaciaacaGaaeqabaqabeGadaaakeaacqWH3bWDdaahaaWcbeqaaiabdsfaubaakiabhIfayjabg2da9maaqadabaGaem4DaC3aaSbaaSqaaiabdMgaPbqabaaabaGaemyAaKMaeyypa0JaeGymaedabaGaemOBa4ganiabggHiLdGccqWGybawdaWgaaWcbaGaemyAaKgabeaaaaa@3E47@ of the species data *X*_1_, ..., *X*_*n *_(see Methods). Thus, in defining our notion of an average, we have also proposed a novel taxon weighting scheme. The implications are explored in subsequent sections.

### Weights and averages

If we restrict our consideration of averages to convex combinations (so-called "weighted averages") **w**^*T*^**X **of the observed data vector **X**, then the assignment of weights to taxa is tantamount to an averaging procedure. The literature on taxon weighting is extensive, particularly with regard to sequence data, and can be coarsely dichotomized into extrapolative and interpolative procedures. The bulk of weighting schemes have been designed to extrapolate beyond the sample; in these methods, weights **w **are chosen to summarize the data **X **so as to maximize the ability of **w**^*T*^**X **to recognize distant similarity to the dataset [5,6,13]. Averaging procedures, by contrast, are interpolative, and seek to summarize the data **X **so as to maximize the similarity between **w**^*T*^**X **and **X **itself. The common thread that unites these weighting schemes is the goal of discounting redundancy due to similarity by descent; however, because the different ends fundamentally require different means, for relevance we limit the discussion to only those interpolative procedures suitable for averaging. Specifically, we will focus on the previously described tree-based Brownian motion approach (henceforth ACL) of [3] and the related distance-based approach (VA) of [10], restricting attention in the latter case to pairwise distances derived from a tree. Both methods seek an interpolative average that mitigates the impact of ancestral relations in an interspecies sample.

### Distances and representative points

**Figure 3:**
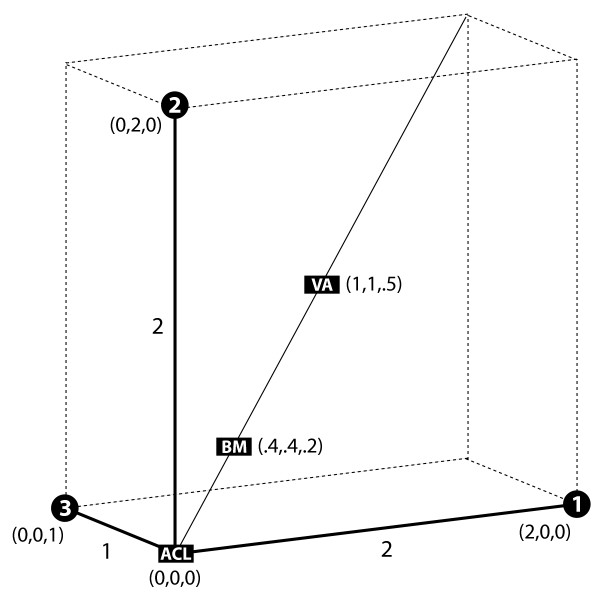
Representative points of a three-taxon tree for various weighting schemes. Solid black lines drawn as *x-*, *y*-, and z-axes show the branches of the tree that join species 1, 2, and 3 (white numbers atop black circles) to the phylogenetic root at (0,0,0). Black rectangles at coordinates (*x*,*y*,*z*) indicate representative points for weighting schemes ACL, BM, and VA.

When considering the taxonomic distribution of an interspecies dataset, there are minimally two issues to consider: (1) the placement of the taxa relative to each other, and (2) the placement of the taxa relative to the evolutionary trajectory described by the tree (see Figure 3). The relative placement of the taxa to each other can be summarized by the matrix **D **of pairwise distances whose entries **D**_ij _record the phylogenetic branch length, and hence the evolutionary divergence, separating species *i *and *j*. The relative placement of the taxa on the tree can be summarized by **D **in concert with an additional vector **z **whose entries **z**_i _record a measure of evolutionary divergence between species *i *and an arbitrary reference that we call the representative point. In the ACL approach, **z**_i _is the divergence (total branch length) between species *i *and the common ancestor at the root at the tree; by contrast, the distance-based VA approach has no explicit representative point and accepts any **z **proportional to the vector **1**. In both cases, the weight vectors can be shown to satisfy the linear relation **Dw **- **z **= *c***1, **where *c *is a normalizing constant so that the weights sum to one. The solution to this equation gives the weights as

w=(1−1TD−1z)D−111TD−11+(1TD−1z)D−1z1TD−1z
 MathType@MTEF@5@5@+=feaafiart1ev1aaatCvAUfKttLearuWrP9MDH5MBPbIqV92AaeXatLxBI9gBaebbnrfifHhDYfgasaacH8akY=wiFfYdH8Gipec8Eeeu0xXdbba9frFj0=OqFfea0dXdd9vqai=hGuQ8kuc9pgc9s8qqaq=dirpe0xb9q8qiLsFr0=vr0=vr0dc8meaabaqaciaacaGaaeqabaqabeGadaaakeaacqWH3bWDcqGH9aqpdaqadaqaaiabigdaXiabgkHiTiabhgdaXmaaCaaaleqabaGaemivaqfaaOGaeCiraq0aaWbaaSqabeaacqGHsislcqaIXaqmaaGccqWH6bGEaiaawIcacaGLPaaadaWcaaqaaiabhseaenaaCaaaleqabaGaeyOeI0IaeGymaedaaOGaeCymaedabaGaeCymaeZaaWbaaSqabeaacqWGubavaaGccqWHebardaahaaWcbeqaaiabgkHiTiabigdaXaaakiabhgdaXaaacqGHRaWkdaqadaqaaiabhgdaXmaaCaaaleqabaGaemivaqfaaOGaeCiraq0aaWbaaSqabeaacqGHsislcqaIXaqmaaGccqWH6bGEaiaawIcacaGLPaaadaWcaaqaaiabhseaenaaCaaaleqabaGaeyOeI0IaeGymaedaaOGaeCOEaOhabaGaeCymaeZaaWbaaSqabeaacqWGubavaaGccqWHebardaahaaWcbeqaaiabgkHiTiabigdaXaaakiabhQha6baaaaa@5952@

which in this form reveals **w **as a convex combination of two weight vectors D−111TD−11
 MathType@MTEF@5@5@+=feaafiart1ev1aaatCvAUfKttLearuWrP9MDH5MBPbIqV92AaeXatLxBI9gBaebbnrfifHhDYfgasaacH8akY=wiFfYdH8Gipec8Eeeu0xXdbba9frFj0=OqFfea0dXdd9vqai=hGuQ8kuc9pgc9s8qqaq=dirpe0xb9q8qiLsFr0=vr0=vr0dc8meaabaqaciaacaGaaeqabaqabeGadaaakeaadaWcaaqaaiabhseaenaaCaaaleqabaGaeyOeI0IaeGymaedaaOGaeCymaedabaGaeCymaeZaaWbaaSqabeaacqWGubavaaGccqWHebardaahaaWcbeqaaiabgkHiTiabigdaXaaakiabhgdaXaaaaaa@3743@ and D−1z1TD−1z
 MathType@MTEF@5@5@+=feaafiart1ev1aaatCvAUfKttLearuWrP9MDH5MBPbIqV92AaeXatLxBI9gBaebbnrfifHhDYfgasaacH8akY=wiFfYdH8Gipec8Eeeu0xXdbba9frFj0=OqFfea0dXdd9vqai=hGuQ8kuc9pgc9s8qqaq=dirpe0xb9q8qiLsFr0=vr0=vr0dc8meaabaqaciaacaGaaeqabaqabeGadaaakeaadaWcaaqaaiabhseaenaaCaaaleqabaGaeyOeI0IaeGymaedaaOGaeCOEaOhabaGaeCymaeZaaWbaaSqabeaacqWGubavaaGccqWHebardaahaaWcbeqaaiabgkHiTiabigdaXaaakiabhQha6baaaaa@3867@ that reflect the taxonomic considerations (1) and (2), respectively. Note that the use of the inverse pairwise-distance matrix in the above formula is what distinguishes the ACL and VA procedures as interpolative and suitable for averaging.

### A framework for comparison

The method we propose, called the BranchManager (BM), can be discussed with ACL and VA in a common framework. Our previously described notion of an average parameter *μ *leads to a weight vector **w **that satisfies the same linear relation **Dw **- **z **= *c***1 **as above (see Methods). The pairwise distance matrix **D **is the same as in ACL and VA; however, the BM vector **z **has entries **z**_i _which record the average divergence between species *i *and any point on the tree. A simple example based on a three-taxon tree helps clarify BM and its relationship to ACL and VA (Figure 3). The tree has been rooted where the three branches meet at an internal node. As mentioned above, the ACL-vector **z **records the branch length between each species and the root; thus, for the example in Figure 3, **z**_*ACL *_= [2 2 1]^*T*^. This can be made more general by embedding the branches of the tree in coordinate space. In Figure 3, the three branches adjacent to species 1, 2, and 3 are respectively embedded into *x*-, *y*-, and *z*-axes with the root of the tree placed at the origin (0,0,0). Species 1, for example, has coordinates (2,0,0) and the divergence between species 1 and the root is computed as the L_1_-distance |2 - 0| + |0 - 0| + |0 - 0| = 2. This is the entry for species 1 in the vector **z**_*ACL*_, and the entries for species 2 and 3 can be similarly expressed; the crux is that the vector **z **can be computed as the L_1_-distance between each species and a representative point, in this case, the phylogenetic root.

Whereas ACL uses an explicit representative point on the phylogeny, the points for VA and BM are implicit and require moving off the phylogeny into extended coordinate space. The representative point for VA is any point L_1_-equidistant to every species on the phylogeny; in the example, this point has coordinates (1,1,.5) and is at an equal distance 2.5 from species 1,2, and 3 (Figure 3). Of course, if the phylogenetic tree is ultrametric so that each species is equidistant from the root, then the points for VA and ACL agree. This reifies the argument of Vingron and Sibbald [13] that ACL and VA weights are the same when the tree is ultrametric; if **D **and **z **are the same, then so too is **w**. By contrast, it is only for a star phylogeny that BM will also in principle agree. The representative point for BM is what we term the "phylogenetic center of mass"; in other words, the balance point of the tree if it were treated as a rigid body. Returning to Figure 3, imagine the tree as three rods, one for each branch, fused together at the origin in space. The center of mass of this object has coordinates (.4,.4,.2), and this acts as the representative point for BM. Crucially, the phylogenetic center of mass adapts to the shape of the phylogeny, whereas the representative points for ACL and VA in general do not. As the following example suggests, it is this quality that lends desirable properties to BM weights and averages.

### A theoretical example

**Figure 4:**
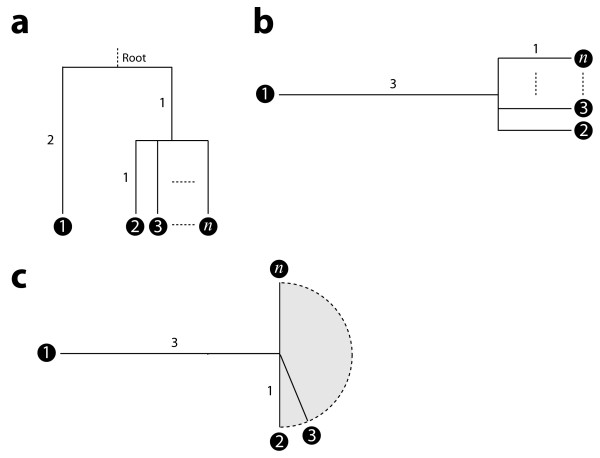
Insufficiency of the root as a reference for an imbalanced tree. (a) The phylogeny relates *n *species, with species 1, 2, 3, and *n *explicitly shown. Branches are drawn to scale, labelled with branch lengths in arbitrary units of evolutionary divergence. "Root" denotes the common ancestor of species 1 through *n *and is equally divergent from all species. Species 2 through *n *share a more recent common ancestor. (b) Unrooted version of the phylogeny in (a). (c) Two-dimensional projection of *n*-dimensional embedding of tree into coordinate space. Shaded grey semicircle is projection of region in which new species are being added.

To illustrate the deficiency of the ACL and VA methods relative to BM, consider a contrived phylogeny of species 1 through *n *(Figure 4a). We have chosen an ultrametric tree so that the methods above can be addressed simultaneously through the *n *× *n *pairwise-distance matrix

D=[044⋯4402⋯2420⋱⋮⋮⋮⋱⋱242⋯20]
 MathType@MTEF@5@5@+=feaafiart1ev1aaatCvAUfKttLearuWrP9MDH5MBPbIqV92AaeXatLxBI9gBaebbnrfifHhDYfgasaacH8akY=wiFfYdH8Gipec8Eeeu0xXdbba9frFj0=OqFfea0dXdd9vqai=hGuQ8kuc9pgc9s8qqaq=dirpe0xb9q8qiLsFr0=vr0=vr0dc8meaabaqaciaacaGaaeqabaqabeGadaaakeaacqWHebarcqGH9aqpdaWadaqaauaabeqafuaaaaaabaGaeGimaadabaGaeGinaqdabaGaeGinaqdabaGaeS47IWeabaGaeGinaqdabaGaeGinaqdabaGaeGimaadabaGaeGOmaidabaGaeS47IWeabaGaeGOmaidabaGaeGinaqdabaGaeGOmaidabaGaeGimaadabaGaeSy8I8eabaGaeSO7I0eabaGaeSO7I0eabaGaeSO7I0eabaGaeSy8I8eabaGaeSy8I8eabaGaeGOmaidabaGaeGinaqdabaGaeGOmaidabaGaeS47IWeabaGaeGOmaidabaGaeGimaadaaaGaay5waiaaw2faaaaa@5179@

Disregarding the root (Figure 4b), the tree has a star-like phylogeny, and it is intuitive that the data from each species should contribute meaningfully to an average in which species 1, owing to its greater divergence, receives proportionately greater weight.

Indeed, both ACL and VA assign species 1 the largest weight n3n−2
 MathType@MTEF@5@5@+=feaafiart1ev1aaatCvAUfKttLearuWrP9MDH5MBPbIqV92AaeXatLxBI9gBaebbnrfifHhDYfgasaacH8akY=wiFfYdH8Gipec8Eeeu0xXdbba9frFj0=OqFfea0dXdd9vqai=hGuQ8kuc9pgc9s8qqaq=dirpe0xb9q8qiLsFr0=vr0=vr0dc8meaabaqaciaacaGaaeqabaqabeGadaaakeaadaWcaaqaaiabd6gaUbqaaiabiodaZiabd6gaUjabgkHiTiabikdaYaaaaaa@3259@; however, the remaining *n*-1 species each receive a paltry 23n−2
 MathType@MTEF@5@5@+=feaafiart1ev1aaatCvAUfKttLearuWrP9MDH5MBPbIqV92AaeXatLxBI9gBaebbnrfifHhDYfgasaacH8akY=wiFfYdH8Gipec8Eeeu0xXdbba9frFj0=OqFfea0dXdd9vqai=hGuQ8kuc9pgc9s8qqaq=dirpe0xb9q8qiLsFr0=vr0=vr0dc8meaabaqaciaacaGaaeqabaqabeGadaaakeaadaWcaaqaaiabikdaYaqaaiabiodaZiabd6gaUjabgkHiTiabikdaYaaaaaa@31E6@. Imagine in the extreme that the datum *X*_1 _from species 1 is an outlier that does not reflect the family, while the data from the remaining species take the identical and biologically interesting value *v*. The ACL and VA averages, assuming the phylogeny of Figure 4, agree and are given by ∑i=1nwiXi=n3n−2X1+2n−13n−2v
 MathType@MTEF@5@5@+=feaafiart1ev1aaatCvAUfKttLearuWrP9MDH5MBPbIqV92AaeXatLxBI9gBaebbnrfifHhDYfgasaacH8akY=wiFfYdH8Gipec8Eeeu0xXdbba9frFj0=OqFfea0dXdd9vqai=hGuQ8kuc9pgc9s8qqaq=dirpe0xb9q8qiLsFr0=vr0=vr0dc8meaabaqaciaacaGaaeqabaqabeGadaaakeaadaaeWaqaaiabdEha3naaBaaaleaacqWGPbqAaeqaaOGaemiwaG1aaSbaaSqaaiabdMgaPbqabaaabaGaemyAaKMaeyypa0JaeGymaedabaGaemOBa4ganiabggHiLdGccqGH9aqpdaWcaaqaaiabd6gaUbqaaiabiodaZiabd6gaUjabgkHiTiabikdaYaaacqWGybawdaWgaaWcbaGaeGymaedabeaakiabgUcaRmaalaaabaGaeGOmaiJaemOBa4MaeyOeI0IaeGymaedabaGaeG4mamJaemOBa4MaeyOeI0IaeGOmaidaaiabdAha2baa@4D10@. As the weight on *X*_1 _is at least one-third, this average can be quite different from *v*, and unfortunately collecting more data will not help: no matter how many new species present data with the value *v*, the contribution of the outlier *X*_1 _to the average will never be less than one-third.

The poor performance of the ACL and VA averages (and hence weights) can be attributed to their common representative point at the root. As more species are added to the phylogeny in Figure 4, the root becomes increasingly unsuitable as the focus for an average. Though the additional lineages contribute evolutionary divergence, this information is effectively ignored at the root; by contrast, the phylogenetic center of mass is adaptive and responds to the addition of branches to the tree. A crude two-dimensional projection of the tree as embedded in *n*-dimensional space (one dimension, or coordinate axis, for each branch) illustrates this point (Figure 4c). The shaded semicircle indicates the region of space in which the branches of additional species are being embedded. The phylogenetic mass in this region is growing with each new lineage, while the mass contributed by species 1 stays constant; thus, as new species are added, the phylogenetic center of mass is being pulled away from species 1 towards the remaining species. This is what we mean by "adaptive": the center of mass responds to the entire evolutionary trajectory.

As the phylogenetic center of mass moves away from species 1, the BM weight on the datum from that species diminishes. Of course, with the addition of more species, the weights on each of the remaining data decrease as well. The weight vector obtained by BM is given by wBMT=[5n−2(n+2)(3n−2)3n+2(n+2)(3n−2)⋯3n+2(n+2)(3n−2)]
 MathType@MTEF@5@5@+=feaafiart1ev1aaatCvAUfKttLearuWrP9MDH5MBPbIqV92AaeXatLxBI9gBaebbnrfifHhDYfgasaacH8akY=wiFfYdH8Gipec8Eeeu0xXdbba9frFj0=OqFfea0dXdd9vqai=hGuQ8kuc9pgc9s8qqaq=dirpe0xb9q8qiLsFr0=vr0=vr0dc8meaabaqaciaacaGaaeqabaqabeGadaaakeaacqWH3bWDdaqhaaWcbaGaemOqaiKaemyta0eabaGaemivaqfaaOGaeyypa0ZaamWaaeaafaqabeqaeaaaaeaadaWcaaqaaiabiwda1iabd6gaUjabgkHiTiabikdaYaqaamaabmaabaGaemOBa4Maey4kaSIaeGOmaidacaGLOaGaayzkaaWaaeWaaeaacqaIZaWmcqWGUbGBcqGHsislcqaIYaGmaiaawIcacaGLPaaaaaaabaWaaSaaaeaacqaIZaWmcqWGUbGBcqGHRaWkcqaIYaGmaeaadaqadaqaaiabd6gaUjabgUcaRiabikdaYaGaayjkaiaawMcaamaabmaabaGaeG4mamJaemOBa4MaeyOeI0IaeGOmaidacaGLOaGaayzkaaaaaaqaaiabl+UimbqaamaalaaabaGaeG4mamJaemOBa4Maey4kaSIaeGOmaidabaWaaeWaaeaacqWGUbGBcqGHRaWkcqaIYaGmaiaawIcacaGLPaaadaqadaqaaiabiodaZiabd6gaUjabgkHiTiabikdaYaGaayjkaiaawMcaaaaaaaaacaGLBbGaayzxaaaaaa@6305@, which is intuitively satisfying. As species 1 remains the most divergent taxon on the star-like tree, its datum receives the largest weight as before. Now, however, the addition of further species is accompanied by a proportionate decrease in all of the weights. Interestingly, the ratio of the weight on *X*_1 _to the each of the remaining weights is w1w2=5n−23n+2
 MathType@MTEF@5@5@+=feaafiart1ev1aaatCvAUfKttLearuWrP9MDH5MBPbIqV92AaeXatLxBI9gBaebbnrfifHhDYfgasaacH8akY=wiFfYdH8Gipec8Eeeu0xXdbba9frFj0=OqFfea0dXdd9vqai=hGuQ8kuc9pgc9s8qqaq=dirpe0xb9q8qiLsFr0=vr0=vr0dc8meaabaqaciaacaGaaeqabaqabeGadaaakeaadaWcaaqaaiabdEha3naaBaaaleaacqaIXaqmaeqaaaGcbaGaem4DaC3aaSbaaSqaaiabikdaYaqabaaaaOGaeyypa0ZaaSaaaeaacqaI1aqncqWGUbGBcqGHsislcqaIYaGmaeaacqaIZaWmcqWGUbGBcqGHRaWkcqaIYaGmaaaaaa@3B77@, which quickly approaches an asymptote of 5/3. That this ratio is stable is indicative of BM's performance; in the cases of ACL and VA, this ratio is n2
 MathType@MTEF@5@5@+=feaafiart1ev1aaatCvAUfKttLearuWrP9MDH5MBPbIqV92AaeXatLxBI9gBaebbnrfifHhDYfgasaacH8akY=wiFfYdH8Gipec8Eeeu0xXdbba9frFj0=OqFfea0dXdd9vqai=hGuQ8kuc9pgc9s8qqaq=dirpe0xb9q8qiLsFr0=vr0=vr0dc8meaabaqaciaacaGaaeqabaqabeGadaaakeaadaWcaaqaaiabd6gaUbqaaiabikdaYaaaaaa@2F13@ and grows unbounded.

### A biological application

**Figure 5:**
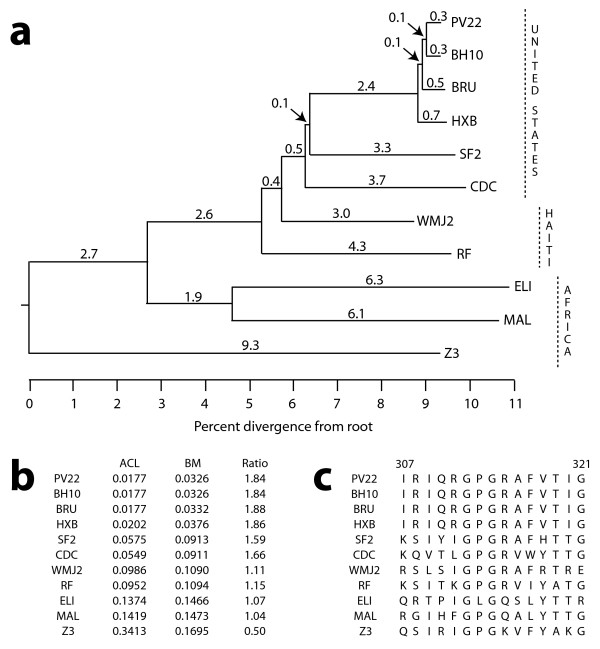
Relationships among eleven HIV-1 isolates. (a) A phylogeny, adapted from [20]. Branches are drawn to scale in units of percent divergence from the root. The isolates were taken from individuals in the United States (PV22, BH10, BRU, HXB, SF2, CDC), Haiti (WMJ2, RF), and Africa (ELI, MAL, Z3). (b) Sets of weights for each of the eleven isolates, calculated from the phylogeny in (a) using ACL (left) and BM (right). Ratio is the BM weight divided by the ACL weight; only Z3 has a ratio smaller than one. (c) Alignment of amino acid positions 307–321 of the HIV-1 envelope glycoprotein gp120 obtained from each of the eleven isolates.

The theoretical situation in Figure 4 is not particularly unrealistic. Real phylogenies are often severely imbalanced, including the very example used to demonstrate the ACL method [3]. We illustrate our method with a subtree of eleven HIV-1 isolates taken from the full phylogeny of fifteen isolates originally presented in [20] (Figure 5a). The isolate Z3 traces its lineage directly to the phylogenetic root; consequently, Z3 carries valuable, non-redundant information about the characteristics of the ancestral virus. The ACL approach reflects this, as the weight of Z3 is 0.3413 (Figure 5b); in other words, focused at the root, Z3 contributes over 34% to the characteristics of the average isolate. But the root may not representative, and in this example most of the diversity is far from the root and Z3. Representation at the phylogenetic center of mass mitigates the influence of Z3, simultaneously rewarding its non-redundancy while penalizing its divergence from the bulk of the remaining isolates [see Additional file 1]. Our approach reduces the weight of Z3 to 0.1695 while upweighting the remaining ten isolates relative to ACL (Figure 5b), leading to an average that better reflects the diversity in the study.

**Figure 6:**
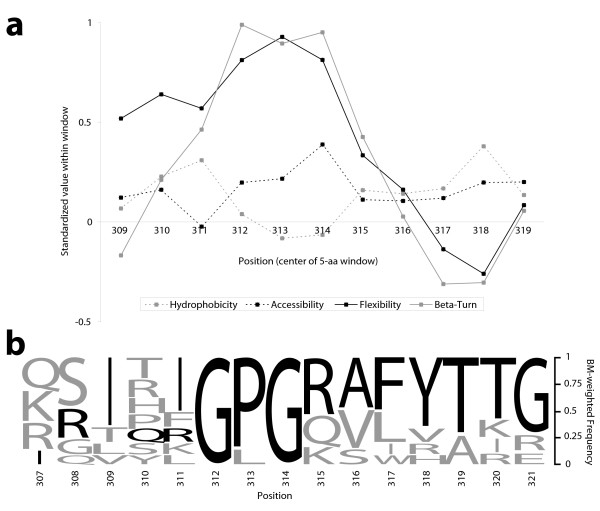
Analysis of eleven HIV-1 isolates. (a) Average physicochemical characteristics of the eleven HIV-1 isolates within the region of gp120 shown in Figure 5c. Four amino acid characteristics are considered: hydrophobicity, surface accessibility, flexibility, and propensity to be in a *β*-turn. Each scale was standardized to facilitate presentation in one plot [see Additional file 2]. Using the BM weights in Figure 5b, four average characteristics were calculated at each position 307–321 in the alignment of Figure 5c. To highlight the trend in these characteristics, the plot displays a moving average (window size 5) across the region. The center of the window is displayed on the *x*-axis, so that 309 corresponds on the *y*-axis to a smoothed value of amino acid positions 307 through 311. High values in the middle of the plot support the recently confirmed hypothesis that positions 312–315 form an exposed *β*-turn. (b) BM-weighted frequency of amino acids by position in the alignment of Figure 5c. For positions 307–321 (left to right), the amino acids in each column are ordered from top to bottom by decreasing frequency after weighting the contribution of each sequence by the BM weights in Figure 5b. By position, the height of each letter shows the weighted frequency of its corresponding amino acid, so that in total all columns have equal height one. The weighted consensus sequence reads across the top as QSITIGPGRAFYTTG, while the standard (unweighted) consensus is given by the sequence of letters in black. Note that the unweighted consensus in position 311 is not unique, as I and R appear four times each.

The significance of choosing appropriate weights to compose an average depends on the characteristics that the average is meant to reflect. In the context of a sample of orthologous proteins, these characteristics are often functions of the amino acid sequences, and in [3] some average properties of the HIV-1 envelope proteins (e.g. number of glycosylation sites, hydrophobicity, predictions of secondary structure) were used to motivate the discussion. As an illustration of this point, consider the alignment of a region of gp120, the major envelope protein of HIV-1 (Figure 5c). Positions 307–321 reside in the variable region V3 of gp120, and have been shown to include the binding site (epitope) for certain type-specific HIV-1-neutralizing antibodies that occur naturally in chimpanzees [21]. The sequence IRIQRGPGRAFVTIG, shared by BH10, PV22, BRU, and HXB, contains a number of amino acids with high *β*-turn potential, hydrophobicity, flexibility, and surface probability [21]. It is natural to ask whether this observation extends to all eleven HIV-1 isolates, or alternatively, whether there are amino acid positions for which the average value of each of these characteristics is high. We therefore turned to a set of scales that quantify each of the aforementioned physicochemical properties [22-25] and then proceeded to compute a BM-weighted average of each at positions 307–321 in gp120 (Figure 5c; see also Additional file 2). Figure 6a shows the results of this analysis. For the sake of presentation, each of the four physicochemical property scales was standardized, and the average characteristics at each position were themselves averaged across a window of width five. This approach highlights the overall trends in the plot, specifically what appears to be an exposed *β*-turn central to the fifteen amino acid stretch (Figure 6a). This echoes the observation first made in [21] and ultimately verified in a subsequent study of the structure of gp120 [26].

The preceding calculations made implicit use of the weighted profile obtained by summing the BM weights of the amino acids present at each position in the alignment of Figure 5c. By design, such a profile has been "corrected" for phylogenetic relationships, resulting in a more faithful representation of the family of sequences comprising the alignment. The BM-weighted profile for positions 307–321 of gp120, presented as a logo [27,28] in Figure 6b, does not resemble the standard profile obtained by assigning each of the eleven sequences equal weight. In particular, because the four most closely-related isolates (PV22, BH10, BRU, HXB) receive only a combined weight of 0.1360, their identical sequence IRIQRGPGRAFVTIG does not overwhelm the BM-weighted profile. Moreover, due to differential treatment of the divergent isolate Z3, the BM-weighted profile also does not resemble the profile obtained by weighting each sequence according to ACL; in the ACL-weighted profile, the sequence QSIRIGPGKVFYAKG from Z3 dominates. The effect of biasing of the profile is most strongly seen through its further compression into a consensus sequence: in Figure 6b, the BM-weighted consensus sequence reads across the top as QSITIGPGRAFYTTG, which disagrees in three of its first four positions with the unweighted consensus sequence shown in black (QSIT vs. IRIQ). Paralleling the theoretical example above, the effect of BM here is to reduce the combined influence of closely-related isolates (e.g. PV22, BH10, BRU, and HXB) without awarding disproportionate weight to highly-divergent ones (e.g. Z3). The result is a profile (and hence consensus) which reflects the "average" sequence over the evolutionary period described by the phylogeny.

## Discussion and conclusion

To function as a useful statistic, an average must faithfully summarize the dataset at hand. When the dataset is comprised of observations from related species, an average should additionally respect the phylogenetic relationships that underlie the data in the study. In this work, we present an alternative to existing methods that naturally achieves both ends. Our approach, the BranchManager (BM), seeks an interspecies average as a parameter *μ *that summarizes the entire evolutionary trajectory relating the data. In doing so, we assume that the sampled species define the taxonomic scope of the study and rely on their phylogeny and a stochastic model to define what the average should be. We derive our estimate of *μ *as a convex combination **w**^*T*^**X **of the species data **X, **and we consider the BM weight vector **w **in a common framework with those obtained by two existing methods: ACL [3] and VA [10]. We show that BM, ACL, and VA each derive their weights using the pairwise-distance matrix **D **obtained from the tree in concert with a unique vector **z **that records the distance between each species and what we call a representative point in extended phylogenetic space. In comparing the representative points used by each method, we explain why ACL and VA may perform poorly for imbalanced phylogenies; conversely, we describe why the phylogenetic center of mass used by BM is adaptive and always reflects the branching structure of the tree. We proceed by example to demonstrate how each representative point influences its respective weights and averages, concluding that those derived by BM exhibit desirable properties.

Because BM uses a representative point that lies off the phylogeny, it is difficult to provide a qualitative description of the weights that it assigns to each species. Informally, our approach uses the dataset to infer its entire evolutionary trajectory, and each species is assigned a weight equal to its contribution to that inference. Thus, the BM weight on a species can be loosely thought of as the fraction of total phylogenetic branch length to which its datum can be in some way attributed. In the two-taxon tree of Figure 1a, this is exactly the case: each species is responsible for half of the length of the branch that connects them, and it is intuitive that each should receive a weight of 0.5. This simple intuition can be extended to a more complicated phylogeny at the cost of some precision. In Figure 5, for example, the isolate Z3 receives a weight of 0.1695, suggesting that it is responsible for 16.95% of the total phylogenetic branch length. The branch length between Z3 and the first divergence point among the remaining isolates is 12.0, and this accounts for 24.69% of the total phylogenetic branch length; half of that percentage, or 12.35%, can be attributed to Z3, making 0.1235 a lower bound for its BM weight. The difference between this lower bound and the true weight of 0.1695 is primarily due to Z3's majority influence on its branch – the taxon across that branch is unobserved, making Z3 the more reliable partner in inferring their common evolutionary trajectory.

Owing to the assumptions underlying its derivation, BM is best suited for averaging quantitative interspecies data. Nevertheless, the weights that our procedure derives can be used to form a weighted consensus of categorical data as well. For example, as an alternative to existing methods of achieving consensus from a family of DNA or protein sequences, BM can be used to define a consensus sequence weighted with respect to the phylogenetic center of mass. For exactly the reasons described in the text, we believe that a profile or consensus formed using BM weights faithfully represents the sequence data and its taxonomic origins. Insofar as BM attempts to bring its average close to the evolutionary path defined by phylogeny, it is not unreasonable to anticipate that a BM-weighted consensus may be a functional member of the sequence family being summarized. Such an approach may also prove fruitful in the area of vaccine design, where consensus, center-of-tree, and ancestral reconstruction methods are already being employed.

With an eye toward the derivation of taxon weights, we have confined the discussion to Brownian motion, but in principle our approach to phylogenetic averaging can be applied to any reversible continuous-time Markov process in which the root position may be ignored. For sequence data, or for functions of sequence data (cf. Figure 6), a suitable model of molecular evolution may be preferable to the Brownian motion we have described [29]. In such an application, because the states of the process are discrete (e.g. nucleotides A, C, G, T), sample paths will be piecewise constant rather than continuous and integrable as in the case of Brownian motion. As a result, sample paths can be summarized by the proportion of time spent in each state, and we can compute the average proportion of time in each state by taking the expectation over all sample paths. In practice, this requires the simulation of many sample paths, from which the average proportion of time in each state can be computed. To clarify this, consider position 307 of the gp120 alignment (Figure 5c). Under a stochastic model for proteins such as JTT [30], we can sample evolutionary paths on the phylogeny that are consistent with the alignment (so that PV22 has an I, Z3 has a Q, etc) and record the proportion of time spent over all paths in each amino acid. This induces a multinomial distribution on the twenty amino acids that can be used to (1) define a consensus, (2) create a profile, or (3) report average characteristics as in Figure 6. Whether such an approach will prove fruitful remains to be seen. The efficacy of our scheme with regard to discrete-state evolutionary processes will be the subject of a future study.

## Methods

### Construction of the weighted average

A flexible approach to the analysis of interspecies data, which we have adopted here, is to model the evolutionary trajectory relating the species data in the study as a continuous-time stochastic process. For our purposes, we consider the tree topology and branch lengths to be known, so that the model resembles a branching process whose branching times are fixed. Just as the leaves of the phylogeny represent extant species with measurable characteristics, the remaining points on the phylogeny represent ancestral species with characteristics of their own. To represent this evolutionary trajectory, we work in a probability space (Ω, *F*, *P*) whose sample paths *ω *∈ Ω are functions defined on the coordinate space defined by the phylogeny. The measure *P *comes from the continuous-time stochastic process and defines a probability on sample paths *ω *∈ Ω, with support confined to those paths which are consistent with the values *X*_1_, ..., *X*_*n *_measured in the extant species. Our notion of an average is defined in terms of these probabilistic sample paths.

The approach of [3] defines an average in terms of the sample paths of a Brownian motion process on the phylogeny directed from the root toward the leaves. Treating the ancestral value at the root as an unknown parameter *μ*, the vector of species data **X **is distributed as multivariate Normal with mean *μ***1 **and a covariance matrix Σ which depends on the phylogeny. For *i*, *j *= 1, ..., *n*, the covariance Σ_*ij *_of *X*_*i *_and *X*_*j *_is equal to the extent of their shared ancestry, calculated as the total branch length between the root and the most recent common ancestor of species *i *and *j*. This also holds when *i *= *j*, in which case the variance of *X*_*i *_is equal to the total branch length between species *i *and the root (see Figure 5a and Additional file 1).

Our approach inverts the reasoning of [3], viewing the species data as fixed and the entire evolutionary trajectory (sample path) as random. We exploit the reversibility of Brownian motion to arbitrarily "root the tree" at one of the extant species (say species 1), thus providing a direction for the stochastic process to flow (see Figure 1). To rectify this with the previous approach, notice that the aforementioned *μ *can now be thought of as *X*_*A*_, the ancestral value of the hypothetical species at the original root of the tree. *X*_*A *_is a random variable obtained by evaluating each sample path *ω *∈ Ω at the phylogenetic root (see Figure 2); its distribution is Normal with mean **w**^*T*^**X **and variance 11TΣ=11
 MathType@MTEF@5@5@+=feaafiart1ev1aqatCvAUfKttLearuWrP9MDH5MBPbIqV92AaeXatLxBI9gBaebbnrfifHhDYfgasaacH8akY=wiFfYdH8Gipec8Eeeu0xXdbba9frFj0=OqFfea0dXdd9vqai=hGuQ8kuc9pgc9s8qqaq=dirpe0xb9q8qiLsFr0=vr0=vr0dc8meaabaqaciaacaGaaeqabaqabeGadaaakeaadaWcaaqaaiabhgdaXaqaaiabhgdaXmaaCaaaleqabaGaemivaqfaaOGaeu4Odm1aaWbaaSqabeaacqGH9aqpcqaIXaqmaaGccqWHXaqmaaaaaa@34A3@, where the weight vector w=Σ−111TΣ−11
 MathType@MTEF@5@5@+=feaafiart1ev1aqatCvAUfKttLearuWrP9MDH5MBPbIqV92AaeXatLxBI9gBaebbnrfifHhDYfgasaacH8akY=wiFfYdH8Gipec8Eeeu0xXdbba9frFj0=OqFfea0dXdd9vqai=hGuQ8kuc9pgc9s8qqaq=dirpe0xb9q8qiLsFr0=vr0=vr0dc8meaabaqaciaacaGaaeqabaqabeGadaaakeaacqWH3bWDcqGH9aqpdaWcaaqaaiabfo6atnaaCaaaleqabaGaeyOeI0IaeGymaedaaOGaeCymaedabaGaeCymaeZaaWbaaSqabeaacqWGubavaaGccqqHJoWudaahaaWcbeqaaiabgkHiTiabigdaXaaakiabhgdaXaaaaaa@3AA3@ is identical to the ACL formulation in [3].

Rather than reduce each sample path *ω *∈ Ω to its value at the phylogenetic root, we obtain an alternative one-dimensional summary ω¯
 MathType@MTEF@5@5@+=feaafiart1ev1aaatCvAUfKttLearuWrP9MDH5MBPbIqV92AaeXatLxBI9gBaebbnrfifHhDYfgasaacH8akY=wiFfYdH8Gipec8Eeeu0xXdbba9frFj0=OqFfea0dXdd9vqai=hGuQ8kuc9pgc9s8qqaq=dirpe0xb9q8qiLsFr0=vr0=vr0dc8meaabaqaciaacaGaaeqabaqabeGadaaakeaaiiGacuWFjpWDgaqeaaaa@2E98@ by integration. To clarify this, note that the unrooted tree *T *corresponding to the phylogeny has 2*n*-3 branches; label these branches *k *= 1, ..., 2*n *- 3 arbitrarily so that branch *k *has length *L*_*k*_. Let L=∑k=12n−3Lk
 MathType@MTEF@5@5@+=feaafiart1ev1aqatCvAUfKttLearuWrP9MDH5MBPbIqV92AaeXatLxBI9gBaebbnrfifHhDYfgasaacH8akY=wiFfYdH8Gipec8Eeeu0xXdbba9frFj0=OqFfea0dXdd9vqai=hGuQ8kuc9pgc9s8qqaq=dirpe0xb9q8qiLsFr0=vr0=vr0dc8meaabaqaciaacaGaaeqabaqabeGadaaakeaacqWGmbatcqGH9aqpdaaeWaqaaiabdYeamnaaBaaaleaacqWGRbWAaeqaaaqaaiabdUgaRjabg2da9iabigdaXaqaaiabikdaYiabd6gaUjabgkHiTiabiodaZaqdcqGHris5aaaa@3B04@ be the total branch length of the tree. We identify the points on branch *k *with the real interval [0, *L*_*k*_], so that every point on the tree can be indexed by a coordinate and its corresponding branch. To parallel the integration in Figure 2, we generatively define a uniform distribution on *T*: choose branch *k *with probability LkL
 MathType@MTEF@5@5@+=feaafiart1ev1aqatCvAUfKttLearuWrP9MDH5MBPbIqV92AaeXatLxBI9gBaebbnrfifHhDYfgasaacH8akY=wiFfYdH8Gipec8Eeeu0xXdbba9frFj0=OqFfea0dXdd9vqai=hGuQ8kuc9pgc9s8qqaq=dirpe0xb9q8qiLsFr0=vr0=vr0dc8meaabaqaciaacaGaaeqabaqabeGadaaakeaadaWcaaqaaiabdYeamnaaBaaaleaacqWGRbWAaeqaaaGcbaGaemitaWeaaaaa@3094@, and then choose a point on that branch by drawing at random uniformly on the interval [0, *L*_*k*_]. In this notation, let *τ *be uniformly distributed on *T*. Then we summarize each sample path *ω *∈ Ω by its average value ω¯
 MathType@MTEF@5@5@+=feaafiart1ev1aaatCvAUfKttLearuWrP9MDH5MBPbIqV92AaeXatLxBI9gBaebbnrfifHhDYfgasaacH8akY=wiFfYdH8Gipec8Eeeu0xXdbba9frFj0=OqFfea0dXdd9vqai=hGuQ8kuc9pgc9s8qqaq=dirpe0xb9q8qiLsFr0=vr0=vr0dc8meaabaqaciaacaGaaeqabaqabeGadaaakeaaiiGacuWFjpWDgaqeaaaa@2E98@ (see Figure 2), computed as the expectation ω¯
 MathType@MTEF@5@5@+=feaafiart1ev1aaatCvAUfKttLearuWrP9MDH5MBPbIqV92AaeXatLxBI9gBaebbnrfifHhDYfgasaacH8akY=wiFfYdH8Gipec8Eeeu0xXdbba9frFj0=OqFfea0dXdd9vqai=hGuQ8kuc9pgc9s8qqaq=dirpe0xb9q8qiLsFr0=vr0=vr0dc8meaabaqaciaacaGaaeqabaqabeGadaaakeaaiiGacuWFjpWDgaqeaaaa@2E98@ = *E*_*τ*_[*ω*(*τ*)].

The probability defined by Brownian motion on the sample paths *ω *∈ Ω induces a probability on ω¯
 MathType@MTEF@5@5@+=feaafiart1ev1aaatCvAUfKttLearuWrP9MDH5MBPbIqV92AaeXatLxBI9gBaebbnrfifHhDYfgasaacH8akY=wiFfYdH8Gipec8Eeeu0xXdbba9frFj0=OqFfea0dXdd9vqai=hGuQ8kuc9pgc9s8qqaq=dirpe0xb9q8qiLsFr0=vr0=vr0dc8meaabaqaciaacaGaaeqabaqabeGadaaakeaaiiGacuWFjpWDgaqeaaaa@2E98@∈ℝ. The mean of this distribution, *μ *= *E*_*P*_[ω¯
 MathType@MTEF@5@5@+=feaafiart1ev1aaatCvAUfKttLearuWrP9MDH5MBPbIqV92AaeXatLxBI9gBaebbnrfifHhDYfgasaacH8akY=wiFfYdH8Gipec8Eeeu0xXdbba9frFj0=OqFfea0dXdd9vqai=hGuQ8kuc9pgc9s8qqaq=dirpe0xb9q8qiLsFr0=vr0=vr0dc8meaabaqaciaacaGaaeqabaqabeGadaaakeaaiiGacuWFjpWDgaqeaaaa@2E98@] = *E*_*P*_[*E*_*τ*_[*ω*(*τ*)]], is our proposed average of averages. Fubini's Theorem permits us to exchange the order of expectation so that *μ *= *E*_*τ*_[*E*_*P*_[*ω*(*τ*)]], and the distribution of *ω*(*τ*) for fixed *τ *is known. To facilitate calculation, let Σ_*τ *_be the covariance matrix which would be obtained under ACL upon rooting the tree at *τ*, and let wτ=Στ−111TΣτ−11
 MathType@MTEF@5@5@+=feaafiart1ev1aqatCvAUfKttLearuWrP9MDH5MBPbIqV92AaeXatLxBI9gBaebbnrfifHhDYfgasaacH8akY=wiFfYdH8Gipec8Eeeu0xXdbba9frFj0=OqFfea0dXdd9vqai=hGuQ8kuc9pgc9s8qqaq=dirpe0xb9q8qiLsFr0=vr0=vr0dc8meaabaqaciaacaGaaeqabaqabeGadaaakeaacqWH3bWDdaWgaaWcbaacciGae8hXdqhabeaakiabg2da9maalaaabaGaeu4Odm1aa0baaSqaaiab=r8a0bqaaiabgkHiTiabigdaXaaakiabhgdaXaqaaiabhgdaXmaaCaaaleqabaGaemivaqfaaOGaeu4Odm1aa0baaSqaaiab=r8a0bqaaiabgkHiTiabigdaXaaakiabhgdaXaaaaaa@4025@ be the corresponding weight vector. Then the distribution of *ω*(*τ*) for fixed *τ *is Normal with mean wτTX
 MathType@MTEF@5@5@+=feaafiart1ev1aaatCvAUfKttLearuWrP9MDH5MBPbIqV92AaeXatLxBI9gBaebbnrfifHhDYfgasaacH8akY=wiFfYdH8Gipec8Eeeu0xXdbba9frFj0=OqFfea0dXdd9vqai=hGuQ8kuc9pgc9s8qqaq=dirpe0xb9q8qiLsFr0=vr0=vr0dc8meaabaqaciaacaGaaeqabaqabeGadaaakeaacqWH3bWDdaqhaaWcbaacciGae8hXdqhabaGaemivaqfaaOGaeCiwaGfaaa@3298@ and variance 11TΣτ−11
 MathType@MTEF@5@5@+=feaafiart1ev1aqatCvAUfKttLearuWrP9MDH5MBPbIqV92AaeXatLxBI9gBaebbnrfifHhDYfgasaacH8akY=wiFfYdH8Gipec8Eeeu0xXdbba9frFj0=OqFfea0dXdd9vqai=hGuQ8kuc9pgc9s8qqaq=dirpe0xb9q8qiLsFr0=vr0=vr0dc8meaabaqaciaacaGaaeqabaqabeGadaaakeaadaWcaaqaaiabhgdaXaqaaiabhgdaXmaaCaaaleqabaGaemivaqfaaOGaeu4Odm1aa0baaSqaaGGaciab=r8a0bqaaiabgkHiTiabigdaXaaakiabhgdaXaaaaaa@3656@ (cf. the results for *X*_*A *_above). Therefore, *μ *= *E*_*τ*_[*E*_*P*_[*ω*(*τ*)]] = *E*_*τ*_[wτTX
 MathType@MTEF@5@5@+=feaafiart1ev1aaatCvAUfKttLearuWrP9MDH5MBPbIqV92AaeXatLxBI9gBaebbnrfifHhDYfgasaacH8akY=wiFfYdH8Gipec8Eeeu0xXdbba9frFj0=OqFfea0dXdd9vqai=hGuQ8kuc9pgc9s8qqaq=dirpe0xb9q8qiLsFr0=vr0=vr0dc8meaabaqaciaacaGaaeqabaqabeGadaaakeaacqWH3bWDdaqhaaWcbaacciGae8hXdqhabaGaemivaqfaaOGaeCiwaGfaaa@3298@] = (*E*_*τ*_[**w**_*τ*_])^*T *^**X**, which once again is a weighted average of the species data *X*_1_, ..., *X*_*n*_. *E*_*τ*_[**w**_*τ*_] is the BM weight vector **w**_*BM *_that was introduced in the Results section.

### Distances and representative points

Because there is a unique path between any two leaves on a tree, the matrix **D **of pairwise distances **D**_*ij *_(total branch length) between species *i *and *j *is uniquely defined and independent of the root position. Nevertheless, the distance matrix **D **relates to the covariance matrix Σ through the representative point at the root. Specifically, let **z **be the column vector whose entries **z**_***i ***_record the distance between each species *i *and the root. Then it is straightforward to deduce that Σ=12[z1T+1zT−D]
 MathType@MTEF@5@5@+=feaafiart1ev1aaatCvAUfKttLearuWrP9MDH5MBPbIqV92AaeXatLxBI9gBaebbnrfifHhDYfgasaacH8akY=wiFfYdH8Gipec8Eeeu0xXdbba9frFj0=OqFfea0dXdd9vqai=hGuQ8kuc9pgc9s8qqaq=dirpe0xb9q8qiLsFr0=vr0=vr0dc8meaabaqaciaacaGaaeqabaqabeGadaaakeaacqqHJoWucqGH9aqpdaWcaaqaaiabigdaXaqaaiabikdaYaaadaWadaqaaiabhQha6jabhgdaXmaaCaaaleqabaGaemivaqfaaOGaey4kaSIaeCymaeJaeCOEaO3aaWbaaSqabeaacqWGubavaaGccqGHsislcqWHebaraiaawUfacaGLDbaaaaa@3DAE@. This gives the relationship between **w**, **D**, and **z**: some algebra verifies that w=Σ−111TΣ−11
 MathType@MTEF@5@5@+=feaafiart1ev1aqatCvAUfKttLearuWrP9MDH5MBPbIqV92AaeXatLxBI9gBaebbnrfifHhDYfgasaacH8akY=wiFfYdH8Gipec8Eeeu0xXdbba9frFj0=OqFfea0dXdd9vqai=hGuQ8kuc9pgc9s8qqaq=dirpe0xb9q8qiLsFr0=vr0=vr0dc8meaabaqaciaacaGaaeqabaqabeGadaaakeaacqWH3bWDcqGH9aqpdaWcaaqaaiabfo6atnaaCaaaleqabaGaeyOeI0IaeGymaedaaOGaeCymaedabaGaeCymaeZaaWbaaSqabeaacqWGubavaaGccqqHJoWudaahaaWcbeqaaiabgkHiTiabigdaXaaakiabhgdaXaaaaaa@3AA3@ satisfies the linear equation **Dw **- **z **= **1**.

Previously we considered rooting the tree at any point *τ *on the tree to obtain a covariance matrix Σ_*τ *_in the spirit of ACL. Defining **z**_*τ *_in analogy to **z **above, Σ_*τ *_can be expressed as Στ=12[zτ1T+1zτT−D]
 MathType@MTEF@5@5@+=feaafiart1ev1aaatCvAUfKttLearuWrP9MDH5MBPbIqV92AaeXatLxBI9gBaebbnrfifHhDYfgasaacH8akY=wiFfYdH8Gipec8Eeeu0xXdbba9frFj0=OqFfea0dXdd9vqai=hGuQ8kuc9pgc9s8qqaq=dirpe0xb9q8qiLsFr0=vr0=vr0dc8meaabaqaciaacaGaaeqabaqabeGadaaakeaacqqHJoWudaWgaaWcbaacciGae8hXdqhabeaakiabg2da9maalaaabaGaeGymaedabaGaeGOmaidaamaadmaabaGaeCOEaO3aaSbaaSqaaiab=r8a0bqabaGccqWHXaqmdaahaaWcbeqaaiabdsfaubaakiabgUcaRiabhgdaXiabhQha6naaDaaaleaacqWFepaDaeaacqWGubavaaGccqGHsislcqWHebaraiaawUfacaGLDbaaaaa@4366@. The significance of this formulation follows from the surprising result that Eτ[1TΣτ−11TΣτ−11]=1T(Eτ[Στ])−11T(Eτ[Στ])−11
 MathType@MTEF@5@5@+=feaafiart1ev1aaatCvAUfKttLearuWrP9MDH5MBPbIqV92AaeXatLxBI9gBaebbnrfifHhDYfgasaacH8akY=wiFfYdH8Gipec8Eeeu0xXdbba9frFj0=OqFfea0dXdd9vqai=hGuQ8kuc9pgc9s8qqaq=dirpe0xb9q8qiLsFr0=vr0=vr0dc8meaabaqaciaacaGaaeqabaqabeGadaaakeaacqWGfbqrdaWgaaWcbaacciGae8hXdqhabeaakmaadmaabaWaaSaaaeaacqWHXaqmdaahaaWcbeqaaiabdsfaubaakiabfo6atnaaDaaaleaacqWFepaDaeaacqGHsislcqaIXaqmaaaakeaacqWHXaqmdaahaaWcbeqaaiabdsfaubaakiabfo6atnaaDaaaleaacqWFepaDaeaacqGHsislcqaIXaqmaaGccqWHXaqmaaaacaGLBbGaayzxaaGaeyypa0ZaaSaaaeaacqWHXaqmdaahaaWcbeqaaiabdsfaubaakmaabmaabaGaemyrau0aaSbaaSqaaiab=r8a0bqabaGcdaWadaqaaiabfo6atnaaBaaaleaacqWFepaDaeqaaaGccaGLBbGaayzxaaaacaGLOaGaayzkaaWaaWbaaSqabeaacqGHsislcqaIXaqmaaaakeaacqWHXaqmdaahaaWcbeqaaiabdsfaubaakmaabmaabaGaemyrau0aaSbaaSqaaiab=r8a0bqabaGcdaWadaqaaiabfo6atnaaBaaaleaacqWFepaDaeqaaaGccaGLBbGaayzxaaaacaGLOaGaayzkaaWaaWbaaSqabeaacqGHsislcqaIXaqmaaGccqWHXaqmaaaaaa@60E7@ (a proof can be found in [31]). Because wBM=Eτ[1TΣτ−11TΣτ−11]
 MathType@MTEF@5@5@+=feaafiart1ev1aaatCvAUfKttLearuWrP9MDH5MBPbIqV92AaeXatLxBI9gBaebbnrfifHhDYfgasaacH8akY=wiFfYdH8Gipec8Eeeu0xXdbba9frFj0=OqFfea0dXdd9vqai=hGuQ8kuc9pgc9s8qqaq=dirpe0xb9q8qiLsFr0=vr0=vr0dc8meaabaqaciaacaGaaeqabaqabeGadaaakeaacqWH3bWDdaWgaaWcbaGaemOqaiKaemyta0eabeaakiabg2da9iabdweafnaaBaaaleaaiiGacqWFepaDaeqaaOWaamWaaeaadaWcaaqaaiabhgdaXmaaCaaaleqabaGaemivaqfaaOGaeu4Odm1aa0baaSqaaiab=r8a0bqaaiabgkHiTiabigdaXaaaaOqaaiabhgdaXmaaCaaaleqabaGaemivaqfaaOGaeu4Odm1aa0baaSqaaiab=r8a0bqaaiabgkHiTiabigdaXaaakiabhgdaXaaaaiaawUfacaGLDbaaaaa@46F7@, the same algebra as before verifies that **w **_*BM *_satisfies the linear equation **Dw **_*BM *_- *E*_*τ*_[**z**_*τ*_] = **1**. By definition, the vector *E*_*τ*_[**z**_*τ*_] records the average distance between each species and any point on the tree; in other words, *E*_*τ*_[**z**_*τ*_] contains the distance between each species and the representative point at the phylogenetic center of mass.

### Implementation

We have shown that the BM average μ=wBMTX
 MathType@MTEF@5@5@+=feaafiart1ev1aaatCvAUfKttLearuWrP9MDH5MBPbIqV92AaeXatLxBI9gBaebbnrfifHhDYfgasaacH8akY=wiFfYdH8Gipec8Eeeu0xXdbba9frFj0=OqFfea0dXdd9vqai=hGuQ8kuc9pgc9s8qqaq=dirpe0xb9q8qiLsFr0=vr0=vr0dc8meaabaqaciaacaGaaeqabaqabeGadaaakeaaiiGacqWF8oqBcqGH9aqpcqWH3bWDdaqhaaWcbaGaemOqaiKaemyta0eabaGaemivaqfaaOGaeCiwaGfaaa@35BF@ can be computed by averaging the weights obtained for all possible rootings of the tree as in **w **_*BM *_= *E*_*τ*_[**w**_*τ*_]. This leads to an economical algorithm because: (1) for each branch, it suffices to compute the weights for just one root position, and (2) the weights for any one root position can be calculated in linear time [3,32]. Combined, this leads to a fast O(*n*^2^) algorithm which we have implemented as a stand-alone Java application. Our algorithm is motivated by the following observation.

Let **w**_*k*,*t *_denote the weight vector obtained by ACL upon rooting the tree on branch *k *at the point indexed by *t *∈ [0, *L*_*k*_]. It is a consequence of Felsenstein's algorithm [32] that 1Lk∫0Lkwk,tdt=wk,Lk/2
 MathType@MTEF@5@5@+=feaafiart1ev1aaatCvAUfKttLearuWrP9MDH5MBPbIqV92AaeXatLxBI9gBaebbnrfifHhDYfgasaacH8akY=wiFfYdH8Gipec8Eeeu0xXdbba9frFj0=OqFfea0dXdd9vqai=hGuQ8kuc9pgc9s8qqaq=dirpe0xb9q8qiLsFr0=vr0=vr0dc8meaabaqaciaacaGaaeqabaqabeGadaaakeaadaWcaaqaaiabigdaXaqaaiabdYeamnaaBaaaleaacqWGRbWAaeqaaaaakmaapedabaGaeC4DaC3aaSbaaSqaaiabdUgaRjabcYcaSiabdsha0bqabaGccqWGKbazcqWG0baDaSqaaiabicdaWaqaaiabdYeamnaaBaaameaacqWGRbWAaeqaaaqdcqGHRiI8aOGaeyypa0JaeC4DaC3aaSbaaSqaaiabdUgaRjabcYcaSmaalyaabaGaemitaW0aaSbaaWqaaiabdUgaRbqabaaaleaacqaIYaGmaaaabeaaaaa@46FF@; this generalizes the progression of Figure 1c and Figure 2, which show the dashed line of means and a subsequent reduction to its midpoint. In particular, Eτ[wτ]=1L∑k=12n−3∫0Lkwk,tdt=∑k=12n−3LkLwk,Lk/2
 MathType@MTEF@5@5@+=feaafiart1ev1aaatCvAUfKttLearuWrP9MDH5MBPbIqV92AaeXatLxBI9gBaebbnrfifHhDYfgasaacH8akY=wiFfYdH8Gipec8Eeeu0xXdbba9frFj0=OqFfea0dXdd9vqai=hGuQ8kuc9pgc9s8qqaq=dirpe0xb9q8qiLsFr0=vr0=vr0dc8meaabaqaciaacaGaaeqabaqabeGadaaakeaacqWGfbqrdaWgaaWcbaacciGae8hXdqhabeaakmaadmaabaGaeC4DaC3aaSbaaSqaaiab=r8a0bqabaaakiaawUfacaGLDbaacqGH9aqpdaWcaaqaaiabigdaXaqaaiabdYeambaadaaeWaqaamaapedabaGaeC4DaC3aaSbaaSqaaiabdUgaRjabcYcaSiabdsha0bqabaaabaGaeGimaadabaGaemitaW0aaSbaaWqaaiabdUgaRbqabaaaniabgUIiYdaaleaacqWGRbWAcqGH9aqpcqaIXaqmaeaacqaIYaGmcqWGUbGBcqGHsislcqaIZaWma0GaeyyeIuoakiabdsgaKjabdsha0jabg2da9maaqadabaWaaSaaaeaacqWGmbatdaWgaaWcbaGaem4AaSgabeaaaOqaaiabdYeambaacqWH3bWDdaWgaaWcbaGaem4AaSMaeiilaWYaaSGbaeaacqWGmbatdaWgaaadbaGaem4AaSgabeaaaSqaaiabikdaYaaaaeqaaaqaaiabdUgaRjabg2da9iabigdaXaqaaiabikdaYiabd6gaUjabgkHiTiabiodaZaqdcqGHris5aaaa@65CD@ so that **w **_*BM *_can be found from the 2*n*-3 weight vectors obtained by rooting the tree at the midpoint of each branch.

### Data and analysis

The phylogeny for the eleven HIV-1 isolates (see Figure 5a) was taken from a larger tree presented in [20]. That manuscript provides details on the isolates and their dates and locations of extraction. The sequence data (see Figure 5c) was given in [21]. To interpret the variation in each alignment column, four physicochemical properties of amino acids were considered: hydrophobicity [24], surface accessibility [23], flexibility [22], and propensity to be in a *β*-turn [25]. To facilitate presentation, each property scale was standardized to have mean zero and unit variance [see Additional file 2]. Let *s*(.) denote one of these standardized scales, and let *A*_*ij *_denote the amino acid in position *j *of isolate *i*, with the isolates ordered from top (*i *= 1) to bottom (*i *= 11) in the alignment of Figure 5c. Let **w **_*BM *_be the BM weight vector in Figure 5b, and let **X**_*j *_= *s*(*A*_.*j*_) be the vector of standardized property values in position *j *corresponding to the amino acids for each isolate *i *= 1, ..., 11. Following the recipe in the text, the average property value (for *s*) at position *j *was calculated as wBMTXj
 MathType@MTEF@5@5@+=feaafiart1ev1aaatCvAUfKttLearuWrP9MDH5MBPbIqV92AaeXatLxBI9gBaebbnrfifHhDYfgasaacH8akY=wiFfYdH8Gipec8Eeeu0xXdbba9frFj0=OqFfea0dXdd9vqai=hGuQ8kuc9pgc9s8qqaq=dirpe0xb9q8qiLsFr0=vr0=vr0dc8meaabaqaciaacaGaaeqabaqabeGadaaakeaacqWH3bWDdaqhaaWcbaGaemOqaiKaemyta0eabaGaemivaqfaaOGaeCiwaG1aaSbaaSqaaiabdQgaQbqabaaaaa@3485@. Trends were emphasized by smoothing these values across the alignment using a window of width five, so that for each property, the moving average 15∑m=−22wBMTXj+m=wBMT∑m=−2215Xj+m
 MathType@MTEF@5@5@+=feaafiart1ev1aaatCvAUfKttLearuWrP9MDH5MBPbIqV92AaeXatLxBI9gBaebbnrfifHhDYfgasaacH8akY=wiFfYdH8Gipec8Eeeu0xXdbba9frFj0=OqFfea0dXdd9vqai=hGuQ8kuc9pgc9s8qqaq=dirpe0xb9q8qiLsFr0=vr0=vr0dc8meaabaqaciaacaGaaeqabaqabeGadaaakeaadaWcaaqaaiabigdaXaqaaiabiwda1aaadaaeWaqaaiabhEha3naaDaaaleaacqWGcbGqcqWGnbqtaeaacqWGubavaaGccqWHybawdaWgaaWcbaGaemOAaOMaey4kaSIaemyBa0gabeaaaeaacqWGTbqBcqGH9aqpcqGHsislcqaIYaGmaeaacqaIYaGma0GaeyyeIuoakiabg2da9iabhEha3naaDaaaleaacqWGcbGqcqWGnbqtaeaacqWGubavaaGcdaaeWaqaamaalaaabaGaeGymaedabaGaeGynaudaaiabhIfaynaaBaaaleaacqWGQbGAcqGHRaWkcqWGTbqBaeqaaaqaaiabd2gaTjabg2da9iabgkHiTiabikdaYaqaaiabikdaYaqdcqGHris5aaaa@544A@ is plotted against each position *j *in Figure 6.

## Availability and requirements

The details of our downloadable Java implementation of the BranchManager are provided below. A script that implements the BranchManager in the statistical language R is available as Additional file 3.

**Project name: **BranchManager

**Project homepage: **

**Operating system: **Unix

**Programming language: **Java

**Other requirements: **Java 1.4 or higher

**License: **GNU GPL

**Any restrictions to use by non-academics: **None

## Authors' contributions

EAS and AS conceived of the study and wrote the paper. EAS developed the theory and analyzed the data. All authors read and approved the final manuscript.

## Supplementary Material

Additional file 1 Two phylogenetic covariance matrices. (a) The ACL covariance matrix Σ under the Brownian motion model for the phylogeny in Figure 5a. The weights in Figure 5b are proportional to **1**^*T*^Σ^-1^. (b) The effective covariance matrix *E*_*τ*_[Σ_*τ*_] used by BranchManager for the same phylogeny. The weights in Figure 5b are proportional to **1**^*T*^(*E*_*τ*_[Σ_*τ*_])^-1^. Click here for file

Additional file 2 Standardized versions of the four physicochemical property scales used in the text. These scales were used in conjunction with the alignment data in Figure 5c to create Figure 6. Click here for file

Additional file 3 An implementation of the BranchManager in the statistical language R. Click here for file
