# Supplementary material for: Constructing a meaningful evolutionary average at the phylogenetic center of mass
Source: BMC Bioinformatics. 2007 Jun 26;8:222. doi: 10.1186/1471-2105-8-222 (PMC1919398; doi:10.1186/1471-2105-8-222)
Supplement: Additional file 1 — Two phylogenetic covariance matrices. (a) The ACL covariance matrix Σ under the Brownian motion model for the phylogeny in Figure 5a. The weights in Figure 5b are proportional to 1TΣ-1. (b) The effective covariance matrix Eτ[Στ] used by BranchManager for the same phylogeny. The weights in Figure 5b are proportional to 1T(Eτ[Στ])-1. [file 1471-2105-8-222-S1.pdf]

a

Covariance matrix Σ under ACL

	PV22	BH10	BRU	HXB	SF2	CDC	WMJ2	RF	ELI	MAL	Z3
PV22	9.2	8.9	8.8	8.7	6.3	6.2	5.7	5.3	2.7	2.7	0.0
BH10	8.9	9.2	8.8	8.7	6.3	6.2	5.7	5.3	2.7	2.7	0.0
BRU	8.8	8.8	9.3	8.7	6.3	6.2	5.7	5.3	2.7	2.7	0.0
HXB	8.7	8.7	8.7	9.4	6.3	6.2	5.7	5.3	2.7	2.7	0.0
SF2	6.3	6.3	6.3	6.3	9.6	6.2	5.7	5.3	2.7	2.7	0.0
CDC	6.2	6.2	6.2	6.2	6.2	9.9	5.7	5.3	2.7	2.7	0.0
WMJ2	5.7	5.7	5.7	5.7	5.7	5.7	8.7	5.3	2.7	2.7	0.0
RF	5.3	5.3	5.3	5.3	5.3	5.3	5.3	9.6	2.7	2.7	0.0
ELI	2.7	2.7	2.7	2.7	2.7	2.7	2.7	2.7	10.9	4.6	0.0
MAL	2.7	2.7	2.7	2.7	2.7	2.7	2.7	2.7	4.6	10.7	0.0
Z3	0.0	0.0	0.0	0.0	0.0	0.0	0.0	0.0	0.0	0.0	9.3

b

Effective covariance matrix under BM

	PV22	BH10	BRU	HXB	SF2	CDC	WMJ2	RF	ELI	MAL	Z3
PV22	8.3	8.0	7.9	7.8	5.4	5.3	5.0	4.6	2.4	2.4	1.9
BH10	8.0	8.3	7.9	7.8	5.4	5.3	5.0	4.6	2.4	2.4	1.9
BRU	7.9	7.9	8.4	7.8	5.4	5.3	5.0	4.6	2.4	2.4	1.9
HXB	7.8	7.8	7.8	8.5	5.4	5.3	5.0	4.6	2.4	2.4	1.9
SF2	5.4	5.4	5.4	5.4	8.8	5.4	5.0	4.7	2.5	2.5	1.9
CDC	5.3	5.3	5.3	5.3	5.4	9.1	5.0	4.7	2.4	2.5	1.9
WMJ2	5.0	5.0	5.0	5.0	5.0	5.0	8.2	4.8	2.6	2.6	2.1
RF	4.6	4.6	4.6	4.6	4.7	4.7	4.8	9.2	2.6	2.7	2.1
ELI	2.4	2.4	2.4	2.4	2.5	2.4	2.6	2.6	11.2	5.0	2.5
MAL	2.4	2.4	2.4	2.4	2.5	2.5	2.6	2.7	5.0	11.1	2.5
Z3	1.9	1.9	1.9	1.9	1.9	1.9	2.1	2.1	2.5	2.5	13.9
