# Supplementary material for: Constructing a meaningful evolutionary average at the phylogenetic center of mass
Source: BMC Bioinformatics. 2007 Jun 26;8:222. doi: 10.1186/1471-2105-8-222 (PMC1919398; doi:10.1186/1471-2105-8-222)
Supplement: Additional file 2 — Standardized versions of the four physicochemical property scales used in the text. These scales were used in conjunction with the alignment data in Figure 5c to create Figure 6. [file 1471-2105-8-222-S2.pdf]

	Hydrophobicity	Accessibility	Flexibility	Beta-Turn
A	0.77	0.61	-0.82	-0.46
C	1.00	-1.56	-0.95	-0.36
D	-1.01	1.03	1.01	1.15
E	-1.01	0.27	0.88	0.10
F	1.10	-0.99	-1.43	-0.91
G	0.03	0.65	1.37	1.72
H	-0.91	-0.95	-1.31	-0.68
I	1.67	-0.84	0.40	-1.11
K	-1.14	2.01	0.52	0.02
L	1.44	-0.08	-0.70	-0.93
M	0.80	-1.52	-1.56	-1.36
N	-1.01	0.65	0.40	0.82
P	-0.37	-0.08	1.01	2.40
Q	-1.01	0.08	0.76	0.07
R	-1.34	-0.19	1.25	-0.18
S	-0.10	1.67	1.01	0.92
T	-0.07	0.76	0.15	0.22
V	1.57	-0.19	-0.46	-1.21
W	-0.14	-1.37	-1.43	-0.48
Y	-0.27	0.04	-0.09	0.25
